# Bovine Papillomavirus Type 2 Infection Associated with Papillomatosis of the Amniotic Membrane in Water Buffaloes (*Bubalus bubalis*)

**DOI:** 10.3390/pathogens9040262

**Published:** 2020-04-04

**Authors:** Valeria Russo, Franco Roperto, Davide De Biase, Pellegrino Cerino, Chiara Urraro, John S. Munday, Sante Roperto

**Affiliations:** 1 Dipartimento di Medicina Veterinaria e Produzioni Animali, Università degli Studi di Napoli Federico II, 80137 Napoli, Italy; valeria.russo@unina.it (V.R.); davide.debiase@unina.it (D.D.B.); chiaraurraro@libero.it (C.U.); 2 Dipartimento di Biologia, Università degli Studi di Napoli Federico II, 80136 Napoli, Italy; roperto@unina.it; 3 Istituto Zooprofilattico Sperimentale del Mezzogiorno, 80055 Portici (NA), Italy; strategia@izsmportici.it; 4 Pathobiology, School of Veterinary Sciences, Massey University, Palmerston North 4410, New Zealand; J.Munday@massey.ac.nz

**Keywords:** amnion, amniotic plaques, bovine papillomavirus type 2, E5 oncoprotein, papilloma, squamous metaplasia, placenta infection, water buffalo

## Abstract

Multiple papillomatous nodules were observed scattered over the amniotic membrane in six water buffaloes that had recently aborted. Grossly, some of the nodules had multiple villous projections while others appeared as single prominent conical or cylindrical horns. Histology revealed folded hyperplastic and hyperkeratotic epithelium supported by a narrow fibro-vascular stalk. Using PCR, sequences of the bovine Deltapapillomavirus type 2 (BPV-2) E5 gene were amplified from the amniotic papillomas. Furthermore, expression of the E5 gene was detected using reverse transcription (RT)-PCR. Western blotting revealed BPV-2 E5 oncoprotein as well as L1 protein, suggesting both abortive and productive infection. Additionally, a functional complex composed of BPV-2 E5 oncoprotein and the phosphorylated PDGFβR was detected, which is consistent with the activation of PDGFβR by the interaction with BPV-2 E5 oncoprotein. These results demonstrate that BPV-2 can infect the amnion of water buffaloes and suggest that this infection may cause proliferation of the epithelial cells of the amnion. While the precise pathogenesis in uncertain, it is possible that BPV-2 infection of stratified squamous epithelial cells within squamous metaplasia foci and/or amniotic plaques could lead to papilloma formation. Papillomavirus-associated amniotic papillomas have not previously been reported in any species, including humans.

## 1. Introduction 

Papillomaviruses (PVs) are small, non-enveloped, double-stranded DNA viruses that infect mucosal and cutaneous epithelia of vertebrates, resulting in benign and malignant lesions of skin and mucosa [1]. More than 200 genotypes of human papillomavirus (HPVs) are fully characterized. Bovine papillomaviruses (BPVs) are the most studied PVs beyond human PVs [2]. Currently, 28 BPVs are fully characterized and classified into five genera: Deltapapillomavirus (δPV) (BPV-1, -2, -13, -14), Xipapillomavirus (χPV) (BPV-3, -4, -6, -9, -10, -11, -12, -15, -17, -20, -23, -24, -26 -28), Epsilonpapillomavirus (εBPV) (BPV-5, -8, -25), Dyokappapapillomavirus (DyoκBPV) (BPV-16, -18, -22), and Dyoxipapillomavirus (DyoχBPV) (BPV-7). BPV-19, BPV-21 and BPV-27 are currently unclassified [3,4]. Bovine δPVs have been associated with a wide range of diseases in cattle as well as in other species. In cattle and water buffalo (*Bubalus bubalis*), they cause cutaneous papillomas and have also been associated with bladder cancer [5,6,7,8,9]. They are the only PVs known to be characterized by a trans-species transmission and infection [1,10]. The bovine δPVs have been associated with sarcoids in horses [11,12], African lions [13], and domestic cats [14]. Additionally, bovine δPV-2 and δPV-13 have been found to cause congenital oral papillomatosis of the lip in newborn lambs [10]. Bovine δPV DNA has also been detected in a squamous cell carcinoma of head and neck in a Connemara mare [15], in a series of cutaneous spindle cell tumors in horses [16], and in skin lesions of Cape mountain zebras, giraffes, and sable antelopes [17]. Like most papillomaviruses, bovine δPV infection can be asymptomatic and DNA has been detected in the skin as well as in the blood of clinically normal cattle. Bovine δPV DNA has also been found in peripheral blood mononuclear cells (PBMCs) of sheep [18], the skin of wild ruminants, and the placenta and blood of mares [19,20]. Furthermore, bovine δPV infection has been found to be associated with skin warts/fibropapillomas [21,22,23], and to play a crucial role in bladder carcinogenesis of cattle and water buffaloes [8]. 

Tumors of the fetal membranes are extremely rare in animals. In cows, placental tumors are limited to a few serendipitous cases of chorangioma [24,25], although the neoplastic nature of this lesion is not universally accepted, being believed to be a tumor-like lesion [26,27]. Amniotic plaques are commonly observed in the placenta of cattle as well as other species. These plaques are 2–4 mm in diameter [28]. They typically appear as a slightly raised annular lesion that histologically appears as a proliferation of hyperplastic epithelium that often shows squamous metaplasia.

In the present paper, multiple amniotic papillomas are described in six water buffaloes. All six buffaloes suffered a late-term abortion of the fetus and the fetal membranes. Molecular methods were then used to demonstrate the presence of bovine δPV-2 infection of the amnion in all six animals. Additionally, the expression of the E5 oncoprotein within the papillomas suggests a potential bovine δPV-2 etiology. 

## 2. Results

### 2.1. Gross and Microscopic Morphology

**Figure 1:**
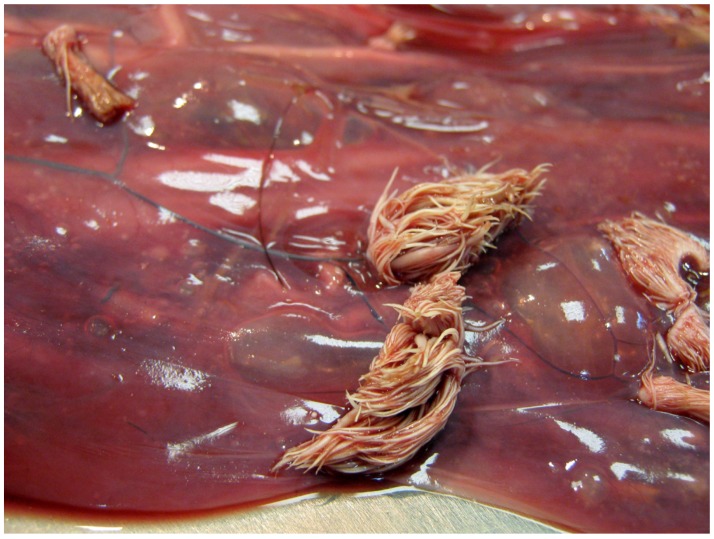
Gross morphology of the amniotic membrane: proliferative tissue lesions diffusely scattered over the amniotic membrane. Some of them appear to be hairy in aspect, like anemone’s fingerlike projections.

**Figure 2:**
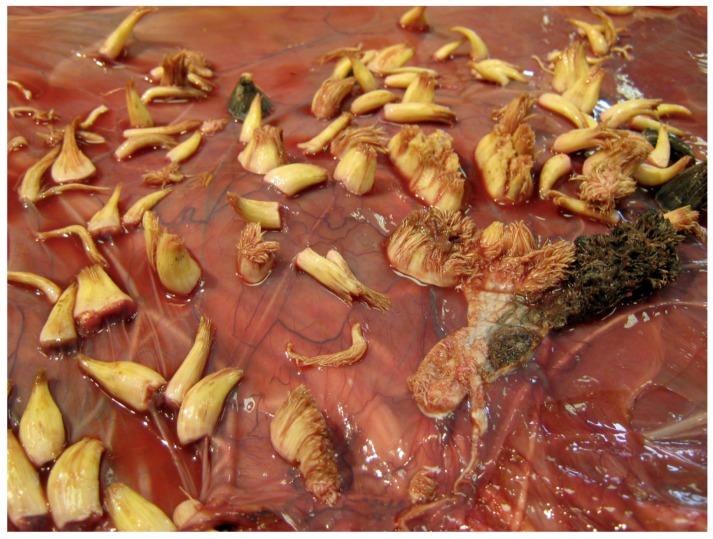
Gross morphology of the amniotic membrane: hard, conical and cylindrical horny proliferations were seen.

Examination of all six affected placentas revealed the presence of between 10 and 30 proliferative lesions up to 3 cm diameter, scattered over the amniotic membrane. Some were filiform with numerous narrow projections while others were horn-shaped or flat (Figure 1 and Figure 2).

**Figure 3:**
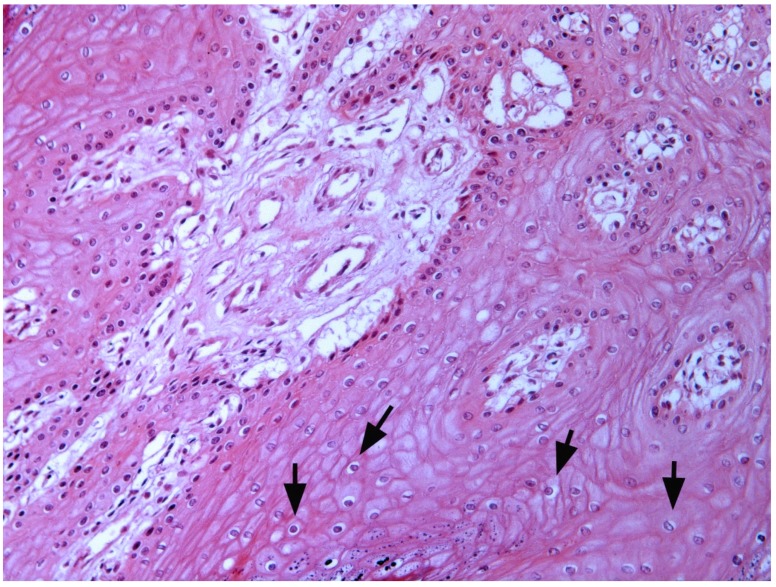
Microscopic pattern of proliferative tissue scattered over the amniotic membrane: The fingerlike projections characterized by a hyperplastic and hyperkeratotic epithelium with a narrow fibro-vascular axis is shown. Many koilocytes were also seen (arrows).

The filiform lesions were soft, while the other lesions were often covered by hard cornified material. Histologically, the fingerlike projections are characterized by hyperplastic and hyperkeratotic epithelium supported by a narrow fibro-vascular core (Figure 3).

**Figure 4:**
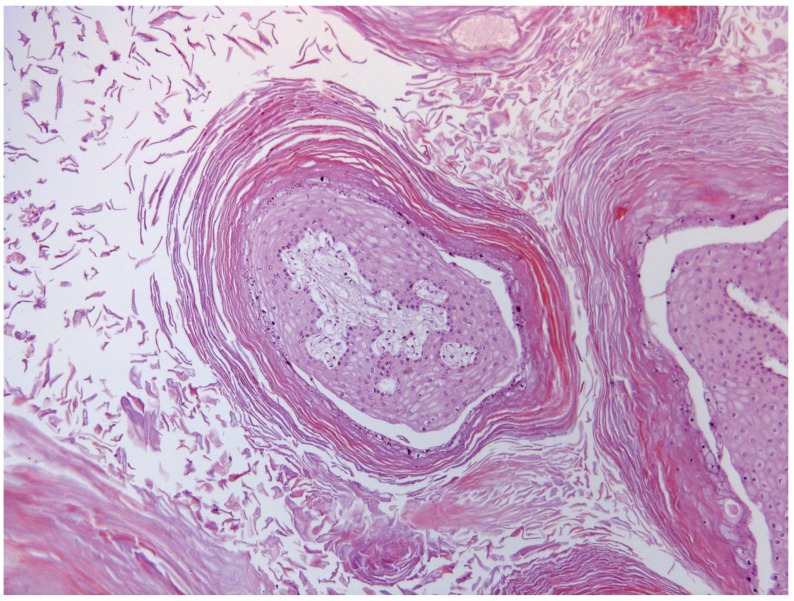
Microscopic pattern of proliferative tissue scattered over the amniotic membrane: A strong orthokeratosis resulting in thick laminated layers of compact anuclear keratin which entirely surrounded tissue proliferation was observed in the stratum corneum of the horny proliferations.

The non-keratinized cells show hydropic vacuolization and intracytoplasmic eleidin granules. The stratum spinosum was hyperplastic and the cells were characterized by large, eosinophilic and vacuolated cytoplasm. Many koilocytes, namely squamous cells, characterized by a sharply defined perinuclear vacuolation, an enlarged nucleus with a prune-like nuclear membrane and a dense-staining peripheral cytoplasm, were seen (Figure 3). In the basal layer, there was moderate mitotic activity. Horn-shaped proliferations were composed of hyperplastic stratified epithelium supported by dermal stalks with dilated capillaries. Marked orthokeratosis was observed in the stratum corneum, resulting in thick laminated layers of compact anuclear keratin overlying the hyperplastic epithelium (Figure 4).

The microscopic patterns are consistent with diagnosis of papillomas with variable orthokeratosis. No papillomatous lesions were visible on examination of any of the 15 placentas from buffaloes that had given birth to a normal calf. However, scattered foci of squamous metaplasia were seen histologically within both the placenta that had papillomas as well as the placenta that had no papillomas. These foci were up to 2mm in diameter and appeared as a thickening of the epithelium up to 10 cells thick, covered by a thick layer of orthokeratosis.

### 2.2. Virological Findings

The presence of koilocytosis was considered as an indication of a possible papillomavirus infection. Papillomavirus DNA was amplified using PCR analysis from all six placentas that had papillomas, but not from the grossly normal placentas.

**Figure 5:**
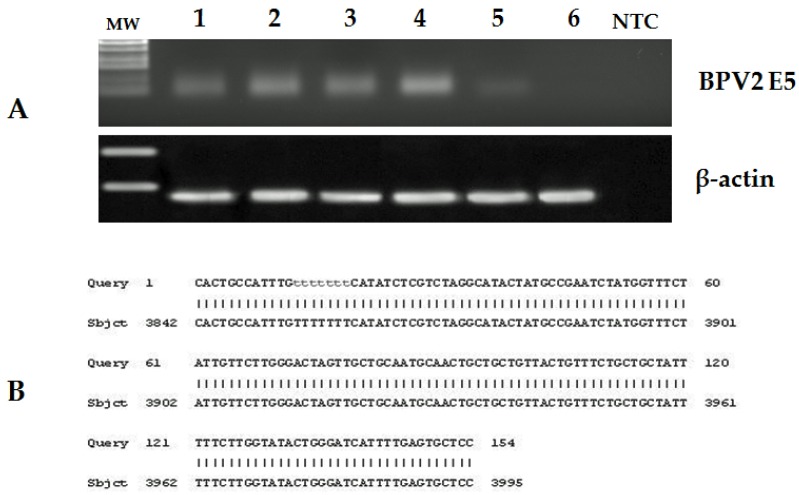
(**A**) PCR of DNA extracted from placentas of pregnant buffalo and a bovine bladder tumor. MW: molecular weight marker; lane 1–4: four neoplastic placentas of pregnant buffalo, lane 5: bovine papillomavirus type 2 bladder tumor (positive control), lane 6: buffalo healthy placenta (negative control), lane NTC: no template control. (**B**) Sequencing results of DNA amplicons. Alignment of the sequences detects a 100% identity with bovine δPV-2 (BPV-2) E5 (Sequence ID: M20219.1).

The 154 bp DNA section amplified is 100% similar to bovine δPV-2 E5 DNA (Accession number: M20219.1) (Figure 5).

**Figure 6:**
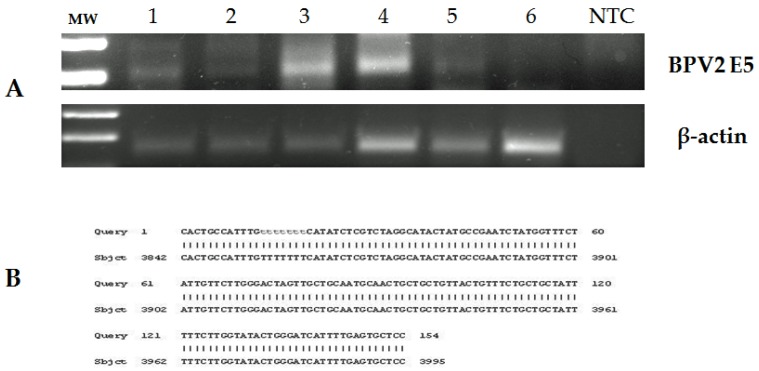
(**A**) RT-PCR: cDNA from RNA extracted from placentas of pregnant buffalo and a bovine bladder tumor. MW: molecular weight marker; lane 1–4 four neoplastic placentas of pregnant buffalo, lane 5: bovine papillomavirus type 2 bladder tumor (positive control), lane 6: buffalo healthy placenta (negative control), Lane NTC: no template control. (**B**) Sequencing results of cDNA amplicons. Alignment of the sequences detects a 100% identity with BPV-2 E5 (Sequence ID: M20219.1).

To evaluate whether or not bovine δPV-2 was transcriptionally active, we investigated the possible presence of E5 transcripts which were found in samples with papillomas by RT-PCR analysis. The amplified cDNA was sequenced and shows a 100% identity with bovine δPV-2 sequences deposited in GenBank (Accession number: M20219.1) (Figure 6).

**Figure 7:**
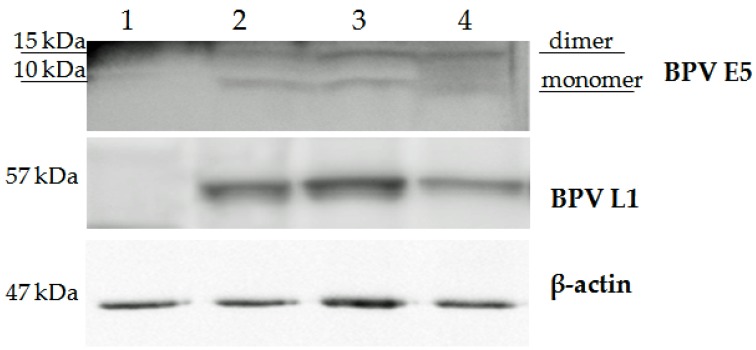
Western blot analysis of BPV E5 (monomer and dimer), L1, and β-actin (as a loading control) in buffalo placentas and bladder tumor tissues. Lane 1: healthy buffalo placenta; lane 2 bovine papillomavirus type 2 bladders tumor; lane 3–4: neoplastic buffalo placenta.

Western blotting confirms the presence of both dimers and monomers of E5 oncoprotein in samples of papillomatous placentas (Figure 7), demonstrating that bovine δPV-2 was transcriptionally active.

**Figure 8:**
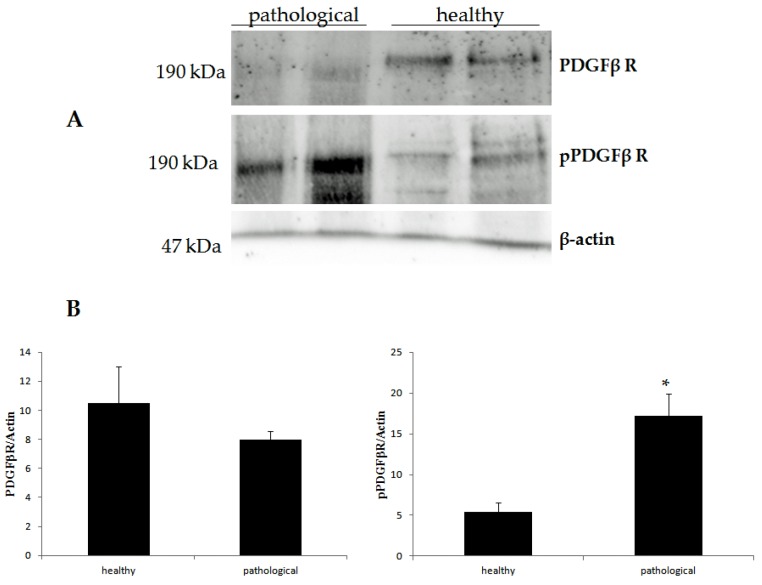
Western blot analysis of total PDGFβR and phosphorylated PDGFβR (pPDGFβR) in healthy and neoplastic buffalo placenta samples. (**A**) Expression of PDGFβR, pPDGFβR and β-actin (as a loading control). (**B**) Densitometric analysis for total PDGFβR and pPDGFβR was performed in comparison with β-actin protein levels. The calculations were based on three independent determinations. The values are expressed as percentages of the average values for the healthy samples (*p < 0.05).

As it has been shown that E5 dimerization is required for transforming activity via phosphorylation of the PDGFβR [29], an immunoblotting investigation to detect total and phosphorylated PDGFβR was also performed. Similar levels of total PDGFβR were detected in both six papillomatous and two grossly normal placentas, whereas there was a statistically significant (*p* ≤ 0.05) increase of phosphorylated PDGFβR in papillomatous placentas compared to the samples of grossly normal placentas (Figure 8). 

The six papillomatous placentas were also shown by Western blotting to contain bovine δPV-2 L1 protein. This protein was not detected from samples of grossly normal placentas (Figure 7). 

### 2.3. Immunohistochemistry 

**Figure 9:**
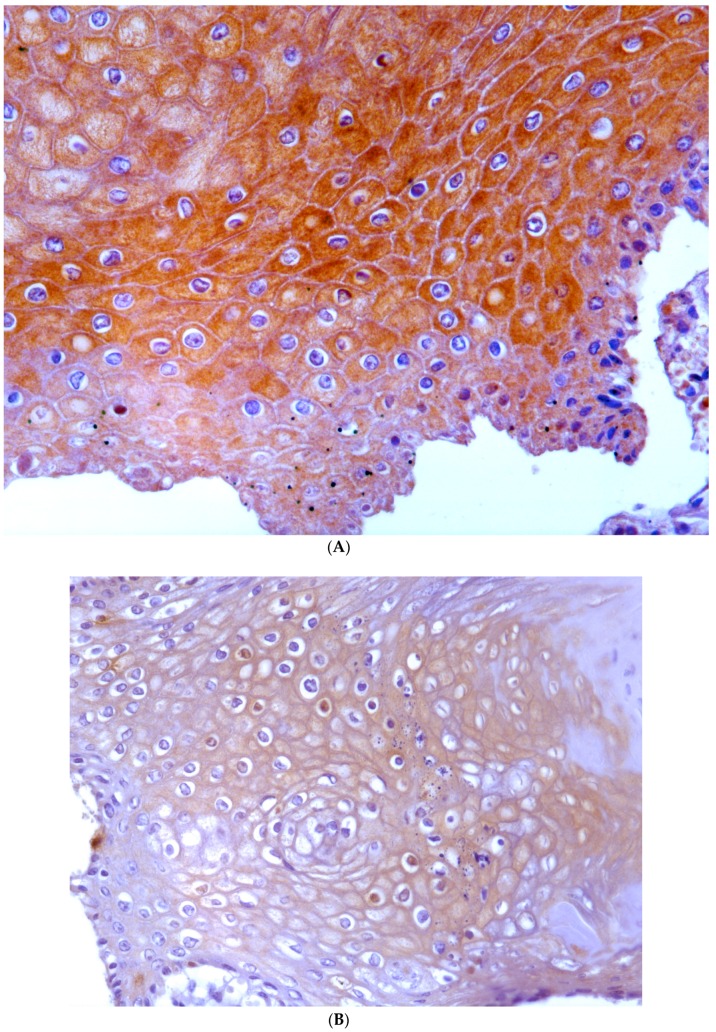
(**A**) Immunohistochemical detection of BPV E5 oncoprotein in hyperplastic epithelium. (**B**) No E5 oncoprotein was detected in control sections from the same pathological tissue samples where the anti-E5 rabbit polyclonal antiserum was omitted and replaced by a normal rabbit serum.

**Figure 10:**
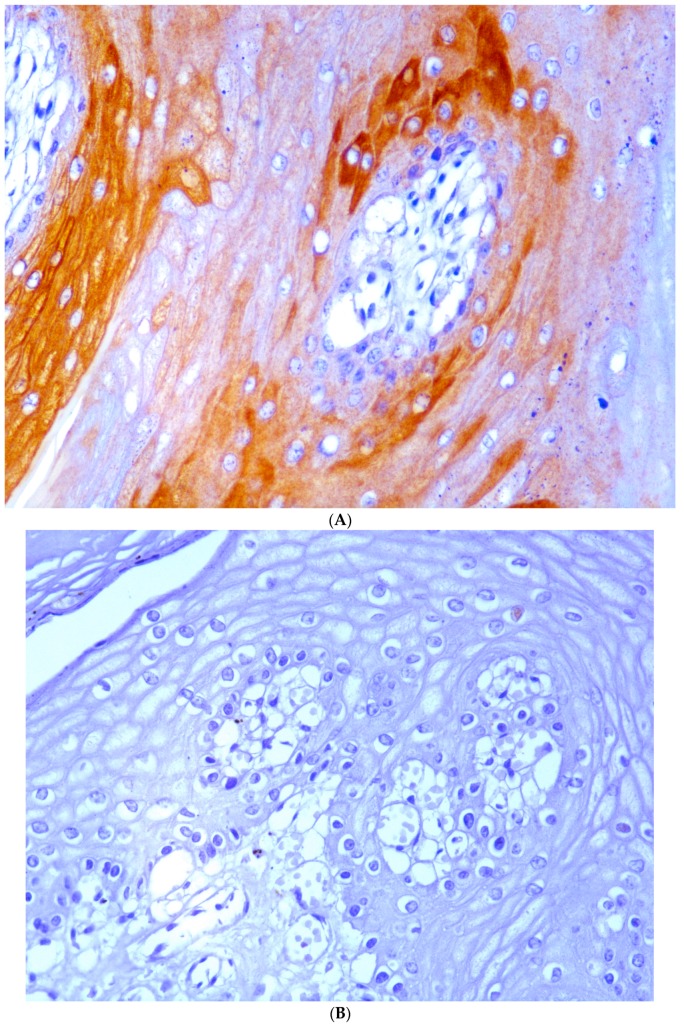
(**A**) BPV L1 structural protein detected in hyperplastic epithelium. (**B**) No L1 protein is shown in hyperplastic epithelium where primary anti-L1 antibody was replaced by appropriate species- and isotype-matched immunoglobulins.

The morphological expression of E5 oncoprotein as well as the structural virus protein L1 was evaluated by immunohistochemistry. In papillomatous placentas, E5 and L1 were detected in the epithelial cells. The most intense E5 immunostaining was seen in the cytoplasm of the basal and suprabasal cell layers (Figure 9A,B). L1 protein was detected in the cytoplasm of the more superficial epithelial layers (Figure 10A,B).

## 3. Discussion

To the authors’ knowledge, this is the first report of multiple large virus-associated papillomas developing on the amnion within any species. The filiform appearance of some papillomas and the presence of marked hyperkeratosis leading to a horn-like appearance of these lesions is consistent with a diagnosis of amniotic squamous papillomas. It is conceivable that these mucosal horn-like lesions may be a variant of mucosal squamous papillomas, just like in humans [30]. Whether or not these lesions were caused by papillomavirus infection cannot be definitively determined. However, cells of all papillomas contained bovine δPV-2 DNA and showed expression of bovine δPV-2 E5 as well as L1 genes. Furthermore, these viral proteins were detected by immunohistochemical investigation. This suggests bovine δPV-2 may have caused the papillomas in these buffaloes. Papillomaviruses have not previously been associated with proliferative placental lesions in domestic species. However, a morphological, purely descriptive study was performed on proliferative lesions observed in the amniotic membrane and umbilical cord of cows [31]. As no virological investigations were performed in the earlier study, it is conceivable that papillomaviruses could be involved in some of the previously described proliferative lesions of the bovine placenta. 

In the present study, the L1 major capsid protein was identified within the papillomas. This protein is only produced late in the viral life cycle and strongly suggests the presence of viral replication within the papillomas. This is the first report of a productive BPV infection of the placenta of buffalo. In addition, the expression and presence of the E5 protein was detected. This protein is thought to promote transforming activity through activation of PDGFβR. Evidence supporting a role of bovine δPV-2 E5 protein in causing the papillomas was derived from detecting higher concentrations of activated (phosphorylated) PDGFβR within the papillomas than within samples of grossly-normal placenta. The presence of bovine δPV-2 E5 and activated PDGFβR has previously been detected in bovine fetuses in which a transplacental transmission of BPV was shown to occur [32] as well as within bovine δPV-2-associated bladder cancers of both cattle and water buffaloes [19,33,34]. Overall, the presence of L1 and E5 proteins supports the hypothesis that the papillomas developed due to the presence of an active bovine δPV-2 infection of the placenta. 

Metaplastic processes are common in the amnion epithelium of women as squamous metaplasia occurs in up to 60% of term placentas. It is believed that foci of squamous metaplasia of the amniotic epithelium do not form in response to chronic inflammation but are found in healthy placentas, thus betraying the maturity of the organs. Indeed, these foci are not seen in immature placentas [35]. 

Like humans, squamous metaplasia is a regular feature of the mature amnion in many other species. It has been found in cattle [31], whales and sheep [35], and dolphins [36]. This study reported the first cases of squamous metaplasia of the bubaline amnion. Cell layers, varying from 3 to 10, were characterized by being structurally similar to mammalian skin epithelium. These metaplastic foci were seen only histologically in the placentas of all fifteen healthy buffaloes examined; therefore, it is conceivable that squamous metaplasia is a physiological feature of mature placentas in water buffaloes. In addition, foci of squamous epithelium composed of numerous cell layers on the internal surface of the amnion have been found to form the so-called amniotic plaques that are apparently normal ill-defined structures, whose physiological function, if any, is unknown [28]. Usually, definitive amniotic plaques are flat in appearance and vary in size from 2 to 4 mm in diameter [28,37]; their height can be up to 8 mm [29]. They have been described in white-tailed deer [37], in cows [38], and in ewes [39,40]. However, it has been suggested that amniotic plaques probably occur throughout eutherian mammals [37].

In the present study, the possibility that the papillomatous lesions represented amniotic plaques was considered. However, the papillomas were much larger than described for amniotic plaques and neither filiform nor horn-covered amniotic plaques have been described. Furthermore, the histological appearance of epithelial folding supported by a thin fibrovascular stalk has not been described as a feature of amniotic plaques. Therefore, the lesions are more consistent with true squamous papillomas rather than abnormally large amniotic plaques. Although the initial development and progression of the papillomas was not investigated, it is possible that foci of squamous epithelial cells harbor BPV DNA and are natural cell sites that may allow the life cycle of BPVs. Infection of the placenta by BPVs has been shown in cattle [41] and transplacental BPV infection has been suggested for several species including sheep [10], cattle [32], and horses [42]. Therefore, it is possible that the foci of squamous metaplasia may allow infection by the BPVs and in the presently described buffaloes this infection may have resulted in papillomatosis of the amniotic epithelium.

All six placenta that had papillomas were from buffaloes that had suffered late-term abortions while no papillomas were observed from buffalo that had carried pregnancies to term. This suggests the development of the papillomas could have caused the abortions. However, it is also possible that changes within the placenta resulted in abortion but also allowed the development of the papillomas. It is worthwhile noting that abnormal activation of PDGFβR by bovine δPV-2 E5 oncoprotein may result in placental abnormalities as it is well known that PDGFβ plays a crucial role in promoting placental angiogenesis and normal placenta development [43,44]; it is also possible that papillomavirus infection may negatively influence placental immune responses. Further cases need to be studied before it can be determined if bovine δPV-2 infection is a cause of abortion in buffalo. 

Finally, a growing body of evidence in comparative medicine suggests that HPVs and BPVs may have an impact on reproductive health and may represent additional infectious pathogens responsible for reproductive disorders. It has been recommended to include HPV testing in the screening for genital tract infections in non-assisted and assisted reproductive technology [45,46]. Cancer and subfertility are considered two sides of the same coin in HPV infection [47]. In veterinary medicine, reproductive disorders like infertility and abortions in large ruminants are major problems and are of high priority in the livestock industry [48]. Indeed, BPV DNA is being found more and more frequently in both the male and female genital systems of large farm animals. Therefore, it would be desirable to screen for the presence of BPV, which could compromise livestock reproduction, in selecting animals for specific commercial purposes. 

## 4. Materials and Methods

### 4.1. Animal and Tissue Samples

Placenta samples were collected from six water buffaloes that suffered from late abortion. The buffaloes came from several farms of Southern Italy. Additionally, samples were collected from fifteen buffaloes that had delivered a normal full-term calf. Parts of the amniotic membranes were immediately fixed in 10% buffered formalin, routinely processed and embedded in paraffin. Histology and immunohistochemistry were performed on 4µm thick sections. Other samples of placentas were frozen in liquid nitrogen and stored at −80°C for subsequent molecular biological analysis. 

### 4.2. Immunohistochemistry

Immunohistochemistry was performed with a rabbit polyclonal anti-E5 antiserum recognizing the C-terminal 14 amino acids of the bovine δPV E5 protein (kind gift of Prof. DiMaio, Yale University School of Medicine, New Haven, CT, USA) and a mouse monoclonal anti-L1 (HPV-16 L1 late protein, Millipore, CA, USA) as previously described [10]. Positive controls were bladder cancer samples where both abortive and productive papillomavirus infection was shown to occur [32]. Antibody specificity was demonstrated by using sections from the same pathological tissue samples where the anti-E5 rabbit polyclonal antiserum was omitted and replaced by a normal rabbit serum (Vector Laboratories Inc., CA, USA), whereas the anti-L1 antibody was replaced by appropriate species- and isotype-matched immunoglobulins(IgGs) (Vector Laboratories Inc., CA, USA) at the same concentration.

### 4.3. DNA Extraction and PCR Amplification

Total DNA was extracted from samples of six bubaline placentas that had papillomas and from samples of two out of 15 healthy placentas as negative controls, using a DNeasy Blood & Tissue Kit (Qiagen TM, DE) according to the manufacturer’s instructions. PCR was performed with 100 ng of DNA. Specific primer sets for PCR were designed by the Primer-BLAST online tool for bovine δPV-2 E5, using β-actin as a control. The following primers were used: bovine δPV-2 E5 forward 5′-TCATAGACATTTGCACGTT-3′, reverse 5′-TCAGGCACAGATCTTGATCA-3′; ß-actin forward 5′-GAGCGTGGCTACAGCTTCA-3′, reverse 5′-CATTGCCGATGGTGATGA-3′. Conditions for PCR were: 94 ℃ for 10 min, 40 cycles of 95 ℃ for 30 s, 56 ℃ for 30 s and 72 ℃ for 30 s. DNA extracted from a papillomavirus-associated bladder cancer sample was used as positive control [9]. 

### 4.4. RNA Extraction and Reverse Transcription (RT)-PCR

Total RNA was extracted from the same samples used for DNA extraction using an RNeasy Blood & Tissue Kit (Qiagen TM, DE) according to the manufacturer’s instructions. Genomic DNA was removed from the RNA samples using RNase-free DNase I from Fermentas Life Sciences (Thermo Fisher Scientific, MA, USA). From the total RNA, 1 µg was used to generate a single strand of cDNA using the QuantiTect Reverse Transcription Kit (Qiagen TM, DE), according to the manufacturer’s instructions. PCR was performed with specific primer sets for the E5 gene of bovine δPV-2, mentioned above, and ß-actin: forward 5′-GAGCGTGGCTACAGCTTCA-3′, reverse 5′-CGGACTCATCGTACTCCTGC-3′. Conditions used for PCR were: 94 °C for 5 min, followed by 40 cycles of 95 ℃ for 30 s, 58 ℃ for 30 s and 72 ℃ for 30 s. RNA extracted from a papillomavirus-associated bladder cancer sample was used as a positive control [9]. 

### 4.5. Sequence Analysis

PCR products from DNA and cDNA were purified using a Qiaquick PCR purification Kit (Qiagen TM, ME, DE), and bidirectionally sequenced using a BigDye_Terminator v1.1 Cycle Sequencing Kit (Applied Biosystems, CA, USA) following manufacturer’s recommendations. Sequences were dye-terminator removed by DyeEx_2.0 spin kit (Qiagen TM, DE) and run on a 3500 Genetic Analyzer (Applied Biosystems, CA, USA). Electropherograms were analyzed using Sequencing Analysis v5.2 and Sequence Scanner v1.0 software (Applied Biosystems, CA, USA). The sequences obtained were compared to others in GenBank using the BLAST program.

### 4.6. Western Blot 

Western blot analysis was performed on two healthy samples as negative controls and six neoplastic full-term placentas of buffaloes. Furthermore, a tissue sample from a bladder cancer with a co-existing abortive and productive BPV infection [9], was used as a positive control for early and late viral proteins. The tissues samples were lysed in RIPA assay-morpholinepropanesulfonic acid (RIPA-MOPS) buffer (20 mM MOPS, 150 mM NaCl, 1 mM EDTA, 1% NP-40, 1% deoxycholate, and 0.1% SDS) containing protease and phosphatase inhibitors and extracted proteins were quantified by Bradford assay. Extracted protein samples of 60 µg were boiled and electrophoresed for 1.5 h at 150 V on an 8–15% (wt/vol) polyacrylamide/SDS gel. Samples were transferred for 1.5 h at 100 V to PVDF membranes in transfer buffer (25 mM Tris base, 192 mM glycine, and 20% (vol/vol) methanol). Membranes were blocked in 5% (wt/vol) nonfat dry milk in TBST (10 mM Tris ⋅ HCl (pH 7.4), 167 mM NaCl, 1% Tween-20) for 1 h and incubated overnight at 4 °C with: anti-E5 rabbit polyclonal antiserum recognizing the C-terminal 14 amino acids of the BPV E5 protein (a kind gift provided by Prof. DiMaio, Yale University, New Haven, USA), an anti-L1 mouse monoclonal antibody (MAB885, Merck Millipore, MA, USA), an anti-PDGFβR rabbit polyclonal antibody (sc-432), an anti-pPDGFβR rabbit polyclonal antibody (sc-1209-R), and an anti-β actin mouse monoclonal antibody (sc-47778) (Santa Cruz Biotechnology, TX, USA). Blots were washed three times in TBST and subsequently incubated for 1 h at room temperature with horseradish peroxidase (HRP)-conjugated donkey anti-rabbit or donkey anti-mouse secondary antibody, diluted 1:3000 in 5% (wt/vol) milk/TBST. Blots were then washed and visualized by enhanced chemiluminescence. 

### 4.7. Statistical Analysis

Results are presented as means ± SE. The expression levels were assessed by one-way Anova, followed by Tukey’s test for multiple comparisons of means, using GraphPad PRISM software version 8 (GraphPad Software, San Diego, CA). *p*-value ≤ 0.05 was considered statistically significant.
